# Multiorgan Failure From Nivolumab and Ipilimumab: A Case Report and Literature Review

**DOI:** 10.7759/cureus.41781

**Published:** 2023-07-12

**Authors:** Danielle Brazel, Seungshin Lee, Aditya Mahadevan, Brian Warnecke, Ritesh Parajuli

**Affiliations:** 1 Department of Medicine, University of California Irvine Medical Center, Orange, USA; 2 Department of Hematology and Oncology, University of California Irvine School of Medicine, Orange, USA; 3 Department of Hematology and Oncology, University of California Irvine Medical Center, Orange, USA

**Keywords:** neutropenic fever, multiorgan failure, immune checkpoint inhibitors, oncology, melanoma, immune related adverse events

## Abstract

Immune checkpoint inhibitors (ICIs) as standard of care have revolutionized the treatment of patients with metastatic melanoma. The combination of nivolumab and ipilimumab improves treatment efficacy and prolongs survival compared to monotherapy alone. However, combination therapy is also associated with an increased incidence of adverse events. We report an uncommon yet important case of multi-organ failure in a patient following a single dose of nivolumab plus ipilimumab.

A 60-year-old male with a history of ulcerative colitis in remission and metastatic melanoma was admitted on February 25, 2021, for presumed sepsis, after presenting with neutropenic fever. His brain metastases were previously resected on January 14, 2021, and he was started on dexamethasone 4 mg BID for six weeks. On February 11, 2021, he received one dose of nivolumab plus ipilimumab, per the CheckMate-067 protocol. He presented 14 days later with fever, diarrhea, pancytopenia, renal failure, drug-induced hepatitis, and myocarditis. The infectious workup was negative. His neutropenia responded to growth factors. He was diagnosed with interstitial nephritis due to immunotherapy and treated with corticosteroids. His symptoms resolved with concomitant improvement of his renal, hepatic, and cardiac function. He was discharged home in a stable condition.

Although these specific immune-related adverse events (irAEs) are uncommon and rarely occur simultaneously, ICIs can trigger non-specific immune system activation, resulting in widespread inflammatory effects. Since irAEs can lead to multi-organ failure, as evidenced in this case, early recognition and institution of high-dose steroids are critical to preventing rapid deterioration. Given that ICI therapy is the standard of care for several cancers and is often studied in clinical trials, increased education on irAE toxicity and updated algorithms on the management of febrile cancer patients are warranted.

## Introduction

The discovery of immune checkpoint inhibitors (ICIs) revolutionized metastatic melanoma treatment and they have become the standard of care. ICIs include anti-cytotoxic T-lymphocyte-associate antigen 4 (CTLA-4) antibodies, such as ipilimumab, and anti-programmed death-1 (PD-1) antibodies, such as nivolumab and pembrolizumab. ICIs are considered first-line therapy for metastatic melanoma and non-small cell lung cancer and are also utilized for Hodgkin's lymphoma and some types of head and neck, bladder, and renal cancer [1]. The results of CheckMate-067 demonstrated that the combination of ipilimumab 3 mg/kg and nivolumab 1 mg/kg improves objective response rate (ORR) and prolongs both progression-free survival and overall survival compared to monotherapy with either drug in unresectable late-stage melanoma [2,3]. However, this combination regimen is associated with increased immune-related adverse events (irAE) [2].

Generally, ICIs are more tolerable with a better safety profile than traditional chemotherapy. Adverse effects of immunotherapy are generally autoimmune-induced inflammation due to their mechanism of action increasing non-specific T cell activity [1]. The most common irAEs include colitis, endocrinopathies, hepatitis, and pneumonitis [2]. Up to 96% of metastatic melanoma patients who receive combination immunotherapy with ipilimumab and nivolumab experienced irAEs, with 59% being Grade 3 or 4 irAE [2,3]. The results of CheckMate-511 demonstrated that the alternative dosing of ipilimumab 1 mg/kg and nivolumab 3 mg/kg significantly reduced the rates of Grade 3 or 4 irAE without inferior ORR, progression-free survival, or overall survival [4]. Although the likelihood of immunotherapy affecting more than one organ is uncommon, patients who received combination immunotherapy experienced significantly higher rates of irAEs compared to single-agent ipilimumab, 31% and 7%, respectively [5]. We present a unique case of multi-organ failure in a patient after receiving a single dose of ipilimumab and nivolumab for the treatment of his metastatic melanoma.

## Case presentation

A 60-year-old male with a history of ulcerative colitis in remission, hypertension, and metastatic melanoma was transferred for presumed sepsis secondary to neutropenic fever. Two years prior to admission, the patient had noticed a purple skin lesion on his right shin, which was subsequently diagnosed as melanoma on biopsy. In addition, a right inguinal lymph node fine needle aspiration (FNA) was also positive for melanoma. PD-L1 was expressed in 5% of tumor cells. The initial staging was consistent with stage IIIC melanoma and was deemed unresectable. A subsequent positron emission tomography (PET) scan confirmed nodal metastases in the right lung and liver. Magnetic resonance imaging (MRI) brain indicated the presence of a mass in the right foramen magnum. MRI spine noted diffuse axial spine lesions. The patient was subsequently started on pembrolizumab and completed 19 cycles until treatment was stopped due to a flare of his underlying ulcerative colitis. His flare was diagnosed clinically on the basis of his worsening diarrhea, but had negative imaging subsequently and was treated with an oral prednisone taper over six months. Post-treatment computed tomography (CT) chest, abdomen, and pelvis revealed stable disease.

He was observed off treatment for about one year, during which he was started on levothyroxine supplementation for ICI-induced hypothyroidism. A surveillance MRI brain obtained one year later showed multiple enhancing masses in bilateral cerebral hemispheres with associated local mass effects. Neurosurgery performed a right frontal lobe mass resection, which was followed by gamma knife radiosurgery two weeks later and dexamethasone therapy for six weeks.

One month following resection, the patient received an initial dose of ipilimumab and nivolumab for the treatment of metastatic melanoma. Two weeks later, he presented to an outside hospital with generalized weakness, poor appetite, shortness of breath, and diarrhea. Initial vitals were significant for a fever of 102.1°F. His complete blood count (CBC) revealed neutropenia with a white blood cell (WBC) count of 0.2 x 10^3^/μL and an absolute neutrophil count (ANC) of 0.005 x 10^3^/μL. He was treated empirically for sepsis, secondary to neutropenic fever, with cefepime and vancomycin and was transferred to our institution for a higher level of care.

On evaluation at our institution, he was afebrile, hemodynamically stable, with an oxygen saturation of 100% on room air. His physical exam was unremarkable. CBC revealed pancytopenia with WBC of 0.2 x 10^3^/μL, hemoglobin of 8.0 g/dL, and platelet count of 93 x 10^3^/μL. Further laboratory workup revealed a creatinine of 4.5 mg/dL (baseline 0.9 mg/dL), glomerular filtration rate (GFR) of 13 mL/min, alkaline phosphatase of 116 IU/L, aspartate aminotransferase (AST) of 17 IU/L, alanine aminotransferase (ALT) of 21 IU/L, total bilirubin of 7.5 mg/dL, direct bilirubin of 5.2 mg/dL, international normalized ratio (INR) of 2.02, brain natriuretic peptide (BNP) of 159 pg/mL, and lactate of 0.9 mmol/L (Table 1). Urinalysis (UA) showed proteinuria. Troponin was elevated to 907 ng/L, and his electrocardiogram (ECG) was normal sinus rhythm, unchanged from the prior.

**Table 1:** Relevant Laboratory Values

	Relevant Laboratory Values										
	Reference Range	Baseline 1/21	Admission 2/25/21	Day 2	Day 3	Day 4	Day 5	Day 6	Day 7	Discharge	One-week Post-discharge
WBC (10^3^/µL)	4-10.5	10.8	0.2	-	0.1	0.2	0.4	1.5	4.6	11.1	-
ANC (10^3^/µL)	2-8.1		15	-	-	-	-	825	3542	7514	-
Hgb (g/dL)	13-16.9	11.5	8.0	-	8.0	8.3	9.1	9.7	9.9	10.1	-
Plt (10^3^/µL)	150-400	181	93	-	94	99	104	100	76	94	-
Cr (mg/dL)	.7-1.3	0.9	4.5	4.3	4.0	3.4	3.3	3.2	2.7	2.3	1.3
GFR (mL/min)	>59	>60	13	14	15	18	19	20	24	29	56
AST (IU/L)	13-39	-	17	15	-	7	7	8	14	12	-
ALT (IU/L)	7-52	-	21	19	-	13	10	11	16	16	-
T. bili (mg/dL)	0-1.4	-	7.5	4.2	-	2.6	2.1	1.9	1.7	1.5	-
D. bili (mg/dL)	0-.2	-	5.2	-	-	1.4	1.2	1.1	0.8	0.7	-
Troponin (ng/L)	0-20	-	-	907	810	208	-	-	-	-	-
INR	.9-1.10	1.13	2.02	2.00	-	1.21	1.19	1.26	1.32	1.21	-
Ferritin (ng/mL)	23-233	-	-	-	-	-	-	178	-	-	-
Fibrinogen (mg/dL)	211-431	-	-	789	-	-	-	543	-	-	-
D-dimer (ng/mL)	<500	-	-	-	-	-	-	420	-	-	-
TG (mg/dL)	<150	-	-	-	230	-	-	-	-	-	-
	WBC: white blood cells, ANC: absolute neutrophil count, Hgb: hemoglobin, Plt: platelets, Cr: creatinine, GFR: glomerular filtration rate, AST: aspartate aminotransferase, ALT: alanine aminotransferase, T. bili: total bilirubin, D. bili: direct bilirubin, INR: international normalized ratio, TG: triglycerides										

His chest X-ray showed an enlarged cardiac silhouette and pulmonary vascular congestion. CT abdomen and pelvis demonstrated colitis of the ascending and sigmoid colon. Abdominal ultrasound showed no evidence of biliary obstruction or common bile duct dilation. Renal ultrasound was unremarkable. Echocardiogram demonstrated a reduced ejection fraction of 46%, globally decreased systolic and diastolic function, and a severely dilated left atrium (Video 1, Table 2). Cardiac MRI showed normal size and morphology of the left ventricle and right ventricle, mild global hypokinesia, and no evidence of abnormal delayed myocardial hyperenhancement to suggest scar or inflammation.

**Video 1:** Transthoracic Echocardiogram Apical four-chamber view revealing mildly decreased left ventricular ejection fraction of 46% with global hypokinesis. RV = right ventricle, LV = left ventricle, RA = right atrium, LA = left atrium

**Table 2:** Two-Dimensional Echocardiogram Read on February 27, 2021

Two-Dimensional Echocardiogram on February 27, 2021	
Left Ventricle	Ejection fraction 46%, global mildly decreased systolic and diastolic function, mild concentric hypertrophy
Right Ventricle	Normal size and systolic function
Left Atrium	Severely dilated
Right Atrium	Normal size and structure
Aortic Valve	Normal structure
Mitral Valve	Trace regurgitation
Tricuspid Valve	Trace regurgitation
Pulmonic Valve	Not well visualized, no evidence of stenosis

Hematology/Oncology was consulted and recommended the initiation of filgrastim and broad-spectrum antibiotics for his neutropenic fever. Nephrology was consulted and deemed his grade 4 acute kidney injury to be secondary to acute interstitial nephritis. A renal biopsy was not performed due to severe thrombocytopenia. For his new onset heart failure, the patient was started on losartan, hydralazine, and furosemide. The patient was started on 125mg IV methylprednisolone for treatment of multiple irAEs, including myocarditis, hepatotoxicity, pancytopenia resulting in neutropenic fever, and nephritis.

Infectious workup, including blood cultures, urine cultures, and stool cultures, remained negative. The patient improved clinically, and antibiotics were discontinued by day 3 of hospitalization. With growth factors, his ANC normalized by hospital day 7. His creatinine improved throughout his hospital course and normalized by follow-up one-week post-discharge (Table 1). The patient's total bilirubin quickly down-trended from 7.5 mg/dL on day 1, to 2.6 mg/dL on day 3 of hospitalization and continued to decline thereafter. His troponin decreased from 907 ng/L on day 1 to 208 ng/L. Following marked clinical and laboratory improvement with high-dose methylprednisolone, he was discharged on hospital day 8 on a prolonged oral prednisone taper for six weeks (Table 1). The patient fully recovered to his baseline two months after discharge.

## Discussion

This case demonstrates a multitude of both common and rare complications of immunotherapy. ICIs can affect virtually all organ systems, though onset and timing vary [6]. Although many of this patient’s irAEs are rare, it is even more uncommon for them to occur simultaneously. The mechanism of irAEs is poorly understood but is thought to involve non-specific T cell over-activation and increased cytokine expression causing a sepsis-like syndrome or aberrant activation of autoreactive T cells [1,7-9]. The combination of nivolumab and ipilimumab is considered first-line therapy in the setting of metastatic melanoma as it prolongs progression-free survival and overall survival. However, it is also associated with increased IrAEs, as seen in our patient [2]. Grade 1 or 2 irAEs can be treated by holding the ICI and administering corticosteroids. The use of systemic, high-dose corticosteroids is essential to reduce the risk of life-threatening complications and progression to organ failure. Some studies have shown that corticosteroids do not impact ICI efficacy even when irAEs occur [7]. Grade 3-4 irAEs often necessitate higher doses of corticosteroids and in some instances, absolute cessation of ICIs.

The diagnosis of irAEs is usually one of exclusion. A full medical workup and appropriate subspecialty consultations are warranted to ensure proper diagnosis. Vigilance was very important for this patient given the use of ICIs with his underlying ulcerative colitis and previous ICI-induced hypothyroidism. Our patient’s presentation is astoundingly unique as he exhibited numerous Grade 3-4 irAEs from a single dose of ipilimumab and nivolumab for his metastatic melanoma, resulting in life-threatening multi-organ failure. From a single dose of ipilimumab and nivolumab, he experienced myocarditis, hepatotoxicity, acute renal failure, and pancytopenia resulting in neutropenic fever. Despite the severity of the patient's presenting symptoms, the neurosurgeon’s recommendation of prolonged dexamethasone, prior to initiation of ipilimumab and nivolumab, may have reduced the severity of the patient's presentation.

The patient's acute renal failure was initially attributed to sepsis; however, given his negative infectious workup and hemodynamic stability, sepsis was deemed unlikely. Nephrology deemed this to be a Grade 4 nephritis secondary to iRAE and a renal biopsy was not performed due to severe thrombocytopenia. Rapid improvement in his serum creatinine, following high-dose corticosteroid administration, further supported the presence of ICI-induced renal injury. Secondly, this patient exhibited Grade 3 hepatotoxicity, as evidenced by significant elevation of his conjugated bilirubin. Although hemophagocytic lymphohistiocytosis was considered as a possible etiology of his liver injury and may result from ICIs, his ferritin, fibrinogen, d-dimer, and triglycerides were only mildly elevated and did not meet the criteria for this diagnosis [10].

Although dermatologic toxicity is the most common irAE in patients with CTLA-4 or PD-1/LD-L1 blockade, this was not demonstrated in our patient [11]. A systematic review reported ICI-induced diarrhea in 27-54% and colitis in 8-22% [12]. The incidence of hepatotoxicity ranges from 3-9% with ipilimumab and 1-2% with nivolumab [13]. Renal irAEs are rare and seen in about 5% of patients on combination ICI regimens [14]. Hematologic involvement is extremely rare and is seen in less than 1% of patients [15]. Hypothyroidism, as seen in our patient, occurs in 6.6% of patients on monotherapy and 13.2% on combination therapy [16].

Myocarditis is a known, rare complication described in 0.06-1% of patients receiving ICI therapy [1,9,17]. In one case series, the majority of patients experiencing cardiotoxicity had prior cardiovascular disease [18]. ECG changes are generally non-specific and highly variable [1]. Biopsy reveals dominant lymphocytic infiltrates with histiocytes (macrophages) in the myocardium with immunohistochemistry staining for CD3 +/CD8+, a few CD4+, and CD68+ cells [1]. Cardiac MRI is the gold standard for non-invasive diagnosis and demonstrates two of the following: (1) increased signal activity on T1- and T2-weighted images reflecting edema; (2) myocardia with more contrast than skeletal muscle reflecting hyperemia; and (3) late contrast enhancement with gadolinium reflecting scar [1]. Although our patient’s cardiac MRI did not meet these diagnostic criteria, we believe that his edema and hyperemia likely resolved from the prior two days of high-dose methylprednisolone. Cardiology deemed this patient’s shortness of breath, troponin elevation, and new cardiac findings on echo and cardiac MRI to be most consistent with ICI-induced myocarditis. Corticosteroids and other immunosuppressive agents are utilized for the treatment of ICI-induced myocarditis. Although multiple second-line agents have been used without evidence, some studies have demonstrated improvement in ejection fraction with corticosteroid treatment [1,18,19].

It is important to note that patients with autoimmune diseases are often excluded from clinical trials involving immunotherapy [17]. Our patient’s history of ulcerative colitis may have placed him at a higher risk compared to patient profiles described in trials and in the literature. His presentation adds to the body of existing literature detailing ulcerative colitis flares when treated with ipilimumab and nivolumab [20]. Further research is needed to evaluate both the efficacy and safety of ICI therapy in patients with autoimmune diseases. Additional research to evaluate drug safety profiles in patients with a history of prior irAEs is needed.

## Conclusions

This patient presented with multiorgan failure after a single dose of ipilimumab and nivolumab. With the increasing utilization of ICIs in many fields within oncology, this case report demonstrates the importance of monitoring these patients for the development of significant irAEs. This requires ongoing vigilance and a thorough work-up, as irAEs are often a diagnosis of exclusion. Often, a multidisciplinary approach, including input from consultants, is necessary to arrive at the proper diagnosis. Following diagnosis, prompt initiation of corticosteroids is essential to prevent rapid deterioration and reduce the likelihood of severe, fatal outcomes.

## References

[1] Atallah-Yunes SA, Kadado AJ, Kaufman GP, Hernandez-Montfort J (2019). Immune checkpoint inhibitor therapy and myocarditis: a systematic review of reported cases. J Cancer Res Clin Oncol.

[2] Larkin J, Chiarion-Sileni V, Gonzalez R (2015). Combined nivolumab and ipilimumab or monotherapy in untreated melanoma. N Engl J Med.

[3] Wolchok JD, Chiarion-Sileni V, Gonzalez R (2017). Overall survival with combined nivolumab and ipilimumab in advanced melanoma. N Engl J Med.

[4] Lebbé C, Meyer N, Mortier L (2019). Evaluation of two dosing regimens for nivolumab in combination with ipilimumab in patients with advanced melanoma: results from the Phase IIIB/IV Checkmate 511 trial. J Clin Oncol.

[5] Hodi FS, Postow MA, Chesney JA (2015). Clinical response, progression-free survival (PFS), and safety in patients (pts) with advanced melanoma (MEL) receiving nivolumab (NIVO) combined with ipilimumab (IPI) vs IPI monotherapy in CheckMate 069 study. J Clin Oncol.

[6] Lomax AJ, McGuire HM, McNeil C (2017). Immunotherapy-induced sarcoidosis in patients with melanoma treated with PD-1 checkpoint inhibitors: case series and immunophenotypic analysis. Int J Rheum Dis.

[7] Weber JS, Dummer R, de Pril V, Lebbé C, Hodi FS (2013). Patterns of onset and resolution of immune-related adverse events of special interest with ipilimumab: detailed safety analysis from a phase 3 trial in patients with advanced melanoma. Cancer.

[8] Nishino M, Sholl LM, Hodi FS, Hatabu H, Ramaiya NH (2015). Anti-PD-1-related pneumonitis during cancer immunotherapy. N Engl J Med.

[9] Kennedy LB, Salama AK (2020). A review of cancer immunotherapy toxicity. CA Cancer J Clin.

[10] Hantel A, Gabster B, Cheng JX, Golomb H, Gajewski TF (2018). Severe hemophagocytic lymphohistiocytosis in a melanoma patient treated with ipilimumab + nivolumab. J Immunother Cancer.

[11] Villadolid J, Amin A (2015). Immune checkpoint inhibitors in clinical practice: update on management of immune-related toxicities. Transl Lung Cancer Res.

[12] Gupta A, De Felice KM, Loftus EV Jr, Khanna S (2015). Systematic review: colitis associated with anti-CTLA-4 therapy. Aliment Pharmacol Ther.

[13] Suzman DL, Pelosof L, Rosenberg A, Avigan MI (2018). Hepatotoxicity of immune checkpoint inhibitors: an evolving picture of risk associated with a vital class of immunotherapy agents. Liver Int.

[14] Cortazar FB, Marrone KA, Troxell ML (2016). Clinicopathological features of acute kidney injury associated with immune checkpoint inhibitors. Kidney Int.

[15] Delanoy N, Michot JM, Comont T (2019). Haematological immune-related adverse events induced by anti-PD-1 or anti-PD-L1 immunotherapy: a descriptive observational study. Lancet Haematol.

[16] Barroso-Sousa R, Barry WT, Garrido-Castro AC, Hodi FS, Min L, Krop IE, Tolaney SM (2018). Incidence of endocrine dysfunction following the use of different immune checkpoint inhibitor regimens: a systematic review and meta-analysis. JAMA Oncol.

[17] Upadhrasta S, Elias H, Patel K, Zheng L (2019). Managing cardiotoxicity associated with immune checkpoint inhibitors. Chronic Dis Transl Med.

[18] Heinzerling L, Ott PA, Hodi FS (2016). Cardiotoxicity associated with CTLA4 and PD1 blocking immunotherapy. J Immunother Cancer.

[19] Escudier M, Cautela J, Malissen N (2017). Clinical features, management, and outcomes of immune checkpoint inhibitor-related cardiotoxicity. Circulation.

[20] Grover S, Ruan AB, Srivoleti P (2020). Safety of immune checkpoint inhibitors in patients with pre-existing inflammatory bowel disease and microscopic colitis. JCO Oncol Pract.

